# Meetings of Societies

**Published:** 1900-03

**Authors:** 


### MEETINGS OF SOCIETIES.

### Bristol Medico-Chirurgical Society.

*December 13th, 1899.*

Mr. W. H. HARSANT, President, in the Chair.

The minutes of the last meeting were read and confirmed. Dr. A. B. McKee, Dr. F. N. Cookson, Dr. W. A Milligan, and Mr. F. P. Mackie were elected members.

Mr. G. MUNRO SMITH showed a patient with syphilitic disease of the elbow of two years' duration. There was anchylosis of the elbow but not of the radio-ulnar joint, and a large mass of new bone had been formed at the lower third of the humerus. A photograph and a skiagram of the patient's arm were shown. He also showed a patient with a marked anterior curvature of both tibiæ, associated with inward rotation of the leg at the knee-joint, which caused a partial dislocation of the patella. He thought the condition was possibly one of achondroplasia rather than of rickets, as there were no signs of the latter disease elsewhere.

Mr. J. PAUL BUSH showed a man suffering from phosphorus necrosis. The patient had worked in a match factory as a dipper, and first noticed his health begin to fail in 1898, when an abscess formed in the upper jaw, discharging some sequestra, probably portions of the floor of the nose. Mr. Bush, after most careful enquiry, was able to exclude syphilis, either congenital or acquired, as a cause. In September the patient got some ulceration round his lower teeth, several of which came out, and in February, 1899, developed septic pneumonia, from which he recovered, only to lose more teeth from further ulceration of the lower jaw. There is now considerable thickening in front of the jaw. Albumin is present in the urine.—Dr. SYMES asked if Mr. Bush had examined the pus for tubercle bacilli, and referred to a paper by Stockman on this matter. (*Brit. M. J.*, 1899, i. 9.)—Mr. ROGER WILLIAMS did not think Mr. Bush had excluded syphilis as a cause, as he found signs of it in nodes on the tibia.—Mr. BUSH stated he had not examined for tubercle bacilli.

Mr. Bush also showed a man, a boxmaker by trade, who had suffered since 1896 from actinomycosis. The first signs were cough, pain in the chest, and a swelling below the left clavicle. It was at first thought this was an empyema pointing, but on opening it the characteristic yellow granules of actinomycosis were seen. Microscopic examination confirmed the diagnosis. Iodide of potassium in drachm doses was given, and though improvement was seen at first, it had not been maintained. Fresh nodules appeared in other places, such as the neck, back, and chest-wall, and in all the man had undergone eight or nine operations for removal.—Mr. Roger Williams spoke of the necessity of recognising these cases, as he believed they were often mistaken for malignant growths, and when cured were reported as successful cases of operations for tumours.

Mr. Carwardine showed a specimen of extra-uterine gestation. On examining the patient, he found displacement of the uterus and a small tumour. At the operation the Fallopian tube opened into a cystic cavity near the uterus, which bled on being opened. The wall of this cyst appeared to be made of organised bloodclot. No fœtus was found. A corpus luteum was observed in the ovary. The patient recovered.—Dr. Aust Lawrence thought the case was one of tubal abortion, and asked if the onset was acute.—Mr. Morton asked if any chorionic villi were observed.—Mr. Carwardine agreed with Dr. Lawrence on the nature of the case, and said that the onset was not acute, the patient suffering for some days from recurrent hypogastric pain. No chorionic villi were seen. He also showed a cyst of the vermiform appendix found while operating on a woman for ovarian cyst. It was as large as a pigeon's egg, and as it could not be emptied he removed it successfully.—Mr. Roger Williams asked whereabouts the constriction was. The fluid in these cases was succus entericus, and such cases were mentioned by Virchow as typical retention cysts.—Mr. Carwardine, in reply, said the constriction was at the middle and terminal third, and he could assign no cause for it.

Mr. Munro Smith related the case of a man, aged 37, who suffered from anthrax. In July, 1899, a cow at the farm on which he worked died after three days' illness and was buried. Early in October another died after two days' illness, and eight days later another, the only symptom being that the beast refused its food. The patient had helped to bury the animals, and said he did not touch them much; but he had a "heat bump" on his wrist, which he scratched as it itched. Next day the arm began to swell and a sore came like a vaccination mark, with a circle of vesicles round it. The swelling extended up the arm, and when Mr. Smith saw him on October 22nd the sore was a depressed slough with dark edges. The whole infected area was removed freely, and the raw surface scraped and rubbed with pure carbolic acid. Numerous incisions were made in the arm and 1 in 20 carbolic acid injected. The man improved and went on satisfactorily till November 6th, when, after the removal of a sloughing tendon from the wound, his temperature rose to 103.5° and continued high for four weeks, when cellulitis of the left leg appeared, and the patient nearly died. Improvement set in again, but the temperature again rose from a gluteal abscess, which was followed by necrosis of the astragalus. After operation improvement was again noticed, and the patient was then in a fair way for recovery. Dr. Symes had verified the diagnosis by culture of pure anthrax. A lantern slide was shown of the growth.—Mr. Morton showed a specimen he had removed that day from the lip of a man who worked in leather. Four days before he had noticed a pimple on his lip, which developed into a pustule the size of a sixpence. There was a dark scab on the surface, and around were small vesicles full of sero-pus. There were no constitutional symptoms. Dr. D. S. Davies, who had seen the case before Mr. Morton, had found anthrax bacilli, and Mr. Morton did not think the diagnosis could have been made from the appearance of the vesicle, though in another case which he had seen it was easy.—Dr. FISHER thought Mr. Morton's case, from his description, presented a typical appearance, and remarked that the œdema, from which it got its name of malignant œdema, was largely dependent on the site, certain spots offering greater facilities for presenting that symptom. He had not heard before of a case in this country in which infection was brought about by direct communication with a diseased animal, it generally coming from hides or wool. —Mr. PAUL BUSH said he reported a few years ago a case in which infection was conveyed directly from the animal to man, while certain pigs which had eaten some of the carcase died of anthrax.—Mr. FIRTH referred to the responsibility that is placed upon a surgeon to decide whether he should operate in a case when no bacilli are found bacteriologically. This had happened to him in two suspected cases, and in both cases he operated, one patient recovering, though in both constitutional symptoms were well marked. He admitted they might neither have been cases of anthrax.—Mr. ROGER WILLIAMS said that during twenty years' hospital experience he had only seen one case of anthrax, and that was at the Bristol Royal Infirmary; and Mr. Dowson operated, recognising the disease immediately. He thought from the number of cases mentioned that it must be fairly common about Bristol.—Mr. MUNRO SMITH, in reply, said no certainty existed in his case till a tube was inoculated, and that the veterinary surgeon, who made no microscopic examination, said it was not anthrax.

Dr. J. O. SYMES read a paper on non-bacillary croup. (Reported on p. 25.)—Dr. THEODORE FISHER referred to some cases recorded by Goodall, and remarked on the clinical similarity of these cases to those of true diphtheria.—The PRESIDENT put the matter the other way, and said it was evident that a form of diphtheria existed of which the Klebs-Löffler bacillus was not the cause.

Dr. BERTRAM ROGERS showed a specimen of sarcomatous growth in the heart removed from a boy, aged 4, who had died at the Children's Hospital. He was admitted in a very collapsed condition, and on examination an enlarged heart, double mitral murmurs, and a small tumour on the fifth right rib were found. The boy contracted diphtheria, which had broken out in the ward, and after 4,500 units of antitoxic serum had been administered he recovered. He died with obscure cerebral symptoms. Optic neuritis was observed before death. At the *post mortem* new growths were found on the ribs, in the kidneys, pancreas, spleen, intestine, root of the left lung, and in the brain. The lower part of the left ventricle was almost entirely replaced by sarcomatous tissue, the heart being much enlarged and displaced.—Mr. ROGER WILLIAMS thought the primary growth must have been on the rib. Secondary sarcomatous growths in the heart were very rare and primary ones even more so. Sarcoma was particularly malignant in children.—Dr. P. WATSON WILLIAMS referred to the importance of examining nasal discharges in children, as he believed diphtheria was often spread by this means.—Dr. THEODORE FISHER referred to certain pericardial growths.

Mr. C. MORTON read a paper on a case of cœliotomy for intestinal obstruction due to a persistent Meckel's diverticulum. The patient was a girl, aged 9 years, who, after five days of vomiting and headache, complained of abdominal pain. When seen by him on the seventh day of illness three coils of distended intestine were visible, but there was no rigidity of the abdomen. The condition suggested appendicitis, and on opening the abdomen some serous fluid escaped, and the small intestine was found distended in parts and collapsed in others. On examination it was found that Meckel's diverticulum, which passed to the umbilicus, had caused strangulation, the small intestine passing below it. The diverticulum was excised. There was a marked groove on the bowel where it had been caught. He inserted a drainage tube, and the patient made a good recovery.—Dr. JAMES SWAIN mentioned a case he had seen recently to illustrate the way in which the intestine is sometimes distended below a complete obstruction. The patient was a woman, aged 42, with acute intestinal obstruction. As he commenced the operation the patient began to vomit, and he had to rapidly complete the operation by bringing a piece of distended bowel to the surface and transfixing the mesentery with a rod. The patient died, and at the *post mortem* a diverticulum three inches long was found, which had been so distended with fæcal matter that it was unable to empty itself, and had so kinked the intestine. The piece of bowel exposed was below the obstruction, and yet was distended.—Mr. ROGER WILLIAMS asked what was the "usual position"—an expression used by the author—of the diverticulum.—Mr. PAUL BUSH asked why Mr. Morton drained.—The PRESIDENT asked what relation did the gut bear to the diverticulum?—Mr. MORTON said that the usual place for the diverticulum was from one to four feet from the ileo-cæcal valve; its position varied between these points. In his case he could not say where it was. He always drained when the gut had been sutured, and had reason to be thankful he had done so on more than one occasion. To the President he replied that he could not speak with confidence: the strangulation was beneath the diverticulum.

---

*January 10th, 1900.*

Mr. W. H. HARSANT, President, in the Chair.

The minutes of the previous meeting were read and confirmed. Mr. Newman Neild, M.B., B.Ch. Vict., was elected a member.

Dr. G. PARKER showed a specimen of aneurysm of the transverse portion of the aorta. The patient was a police inspector, aged 40, and he had been under observation for three years or more. He dated his troubles from an attack of influenza; but at no time were his symptoms characteristic of aneurysm. He had some inspiratory dyspnœa, and the left vocal cord became paralysed; there were no tubercle bacilli in the sputum. Later on in the disease the left radial pulse became almost obliterated, and there was dulness in the second and third left intercostal spaces, with some pulsation in the first three. Before death, on December 10th, the dyspnœa diminished. At the autopsy the aneurysm shown was discovered pressing on the left bronchus; it was when fresh the size of two fists, and contained some laminated clot. There was slight atheroma of the aorta. One rib cartilage was much eroded. The "posticus" muscle of the left side was degenerated. The other organs were healthy.—Dr. P. WATSON WILLIAMS asked whether the aneurysm pressed on the vagus or recurrent laryngeal. When he examined the patient about three years ago he found abductor paresis and some downward displacement of the heart.—Dr. T. FISHER enquired if there were any signs of venous congestion to distinguish it from a case of mediastinal growth; also was there any tracheal tugging?—Mr. Firth asked if flattening of the trachea was present when the specimen was fresh.—Dr. G. Parker, in reply, said he was able to answer all the questions in the negative, except Dr. Williams', to which he could not reply.

Dr. T. Fisher showed a specimen of hemorrhage into the intestine of a child. The hemorrhage was beneath the mucous membrane, and extended from the hepatic flexure to the transverse colon. There was no thrombosis of any vessel. In microscopic sections of the liver he had found small thrombi in the portal vein, but these he thought were secondary. Dr. Stack took the patient in twice to the Infirmary. On the first occasion he was put in a surgical ward, as it was thought at first that the great distension of the abdomen was due to obstruction. He was, however, soon moved to a medical ward, and as he got better he was discharged. No definite diagnosis was made, and there was no hemorrhage from the bowel. He came in again two months later, having been ill acutely for one day. He was then very ill and had a "peritoneal" aspect, suffering from constant and excessive attacks of great abdominal pain. After three days he died. Dr. Fisher also showed a specimen of congenital dilatation of the colon from a child six months old; an ulcer in a portion of a strangulated small intestine, not unlike a typhoid ulcer; also a large spleen and liver; and a heart showing fibroid disease.—Mr. Munro Smith and Dr. Michell Clarke thought the large liver and spleen were due to syphilis; the latter had seen two cases of hemorrhage into the intestine like that shown.—Dr. Fisher did not agree with Mr. Smith or Dr. Clarke: the syphilitic theory did not explain the enlarged spleen.

The President showed a large fibroid tumour he had enucleated. A clamp had to be applied to arrest the hemorrhage.

Dr. Aust Lawrence read a paper on the treatment of urethral caruncle. (Reported on p. 23.)—Dr. W. C. Swayne agreed that recurrence was rare if the removal was complete. Some cases simulated malignant disease, and he mentioned a case in which the sections of the caruncle looked exactly like epithelioma. Though the operation was done some years ago, the patient is quite well.—Dr. Newnham related a case in which the patient suffered great pain on coitus; he removed the caruncle, and the symptoms were relieved.—Mr. J. Ewens advocated total removal of the growth.—Dr. Stack asked whether Dr. Lawrence recognised any difference between the red and blue varieties of the growth, the red ones, in his experience, being more sensitive and more likely to recur.—The President remarked that partial removal often leads to hemorrhage. He narrated a case in which this occurred several days after operation, the hemorrhage being stopped by complete removal.—Dr. Lawrence, in reply, advocated complete removal, especially with the blue ones, which he thought were nearly allied to a varicose condition.

Dr. W. C. Swayne read some notes on a case of symphysiotomy. This was his second case; unfortunately, the child died. The patient was a woman aged 45, and a primipara, having been married six years. She was 5 ft. 1 in. in height, and did not appear to be abnormally small. Soon after labour commenced the membranes ruptured. The following measurements were taken:—Between anterior sup. spines of ilium, 9½ inches; between most distant parts of iliac crests, 10½; ext. conj., 7¼; diag. conj. 3½; int. conj., estimated 2¾. At 10.30 he operated, but the symphysis did not separate well, the shape of the joint necessitating section from both above and below. On the section being completed there was considerable hemorrhage, which required plugging, and as the os was not fully dilated, he put in a de Ribes' bag. After some time he was able to apply axis-traction forceps; but in delivering the head the cervix and perineum were lacerated. The child did not breathe in spite of all efforts, though the heart was beating at the time of birth. The wound was dressed and the patient returned to bed. On the second day she tore off all her dressings, which performance she repeated on the fifth day; and as the wound in consequence became septic, the stitches were removed. Staphylococci were grown from the scrapings from the skin. She then developed pneumonia, probably septic in character, and on recovering from this she had one small rise of temperature before she went home convalescent, with only a slight uterine displacement.—Mr. BUSH said that he was present, and was surprised at the very little amount of separation of the bones. He thought valuable time might have been saved if Dr. Swayne had used the big bone forceps.—Dr. LAWRENCE spoke of the many difficulties in the case, and thought that possibly the absence of separation might have been due to some old disease in the sacro-iliac joint. He did not recommend the use of bone forceps suggested by Mr. Bush, for as a rule there was only cartilage to cut. Had the woman been seen earlier, labour might have been induced.—Dr. J. G. SWAYNE said that want of success formerly was due to ignorance of antiseptic treatment. He thought the death of the child was due to the want of separation of the bones. —Dr. W. C. SWAYNE, in reply, said he was tempted to use the bone forceps, but he did not wish to open the cancellous bone. The difficulty in section arose from the fact that the union instead of being straight was of an obtuse-angled V-shape, the upper and lower parts sloping towards the left.

B. M. H. ROGERS, *Reporter.*
J. PAUL BUSH, *Hon. Secretary.*

---

*February 14th, 1900.*

Mr. W. H. HARSANT, President, in the Chair.

The minutes of the last meeting were read and confirmed. Dr. G. H. Barker and Mr. E. W. H. Groves were elected members of the Society.

Mr. T. CARWARDINE showed the following specimens :—(1) A "loose body" he had removed from the knee of a man 32 years of age. The patient, during the six months previous to operation, had had recurring attacks of synovitis, and had detected the loose body himself in that time. Mr. Carwardine, in defiance of the rule against the practice, had in this case opened the joint without immediately before feeling the "loose body." The "body" was a piece of cartilage with a spicule of bone attached on one side. It had obviously been detached from the internal condyle, for a recess was found in that condyle of corresponding size. A case of Lane's was alluded to, in which similar bodies, though of pure cartilage, had been removed from each knee of a patient, and in that case symmetrical punched-out cavities had been seen, showing where the separation had taken place. (2) A "loose cartilage" removed from the knee. The patient, a man weighing seventeen stone, had suffered repeatedly from severe attacks of synovitis during the eight months preceding the operation. The loose cartilage was, as usual, the inner one. Complete recovery had followed its removal. (3) An ivory exostosis removed from the left nasal fossa. The growth had been detached from its connections in the nasal fossa with a narrow saw passed through the left nostril. For its removal it was necessary to reflect up the nose and upper lip by Rouge's method, and chisel off a portion of the margin of the anterior nares. The weight of the specimen was 560 grains.—Dr. WATSON WILLIAMS remarked that such nasal growths as that described, like naso-pharyngeal polypi, occurred in young males especially. They had no tendency to disappear spontaneously. The case illustrated how misleading rhinoscopic appearances might be, for only the anterior end of the growth could be seen, and no indication was given of the large size of the growth. Similar optical illusions were sometimes produced in laryngoscopical examinations. The nature of enchondromata and bony growths was revealed by the probe. They were not likely to recur after removal.

Dr. E. C. WILLIAMS showed a case of the Mongolian type of imbecile. He remarked that the case was a fairly typical one of its kind. The term "Mongolian" had been first used by Langdon Down in his classification of idiots on an ethnological basis. The child was five years old. As an infant he had had snuffles and sores on the buttocks. He did not learn to sit up until he was three years old, and now at five could only just stand. The head was remarkably brachycephalic, appearing as if it had been compressed antero-posteriorly. The palpebral fissures were somewhat oblique, and the eyelids drooped. The tongue protruded slightly. The skin was soft, the hair not coarse. There was a mitral systolic heart murmur. The fingers were rather thick, but tapered at the tips. The index and little fingers seemed dwarfed as compared with the others. The child almost constantly smiled, and made grimaces. The distinctive characteristics of the Mongolian idiot and the Cretin were pointed out. The active movements of the limbs and face in the Mongol contrasted with the lethargic demeanour of the Cretin. In the latter also there were no characteristic skull changes, the relative size of the fingers was normal, the thyroid gland absent, the hair coarse and scanty, and there were fatty deposits in the neck. Thyroid extract was of great benefit to the Cretin, but in the Mongol had no effect. Dr. Wilmarth (*Alienist and Neurologist*, October, 1890) had given an account of five autopsies on Mongolian idiots, and had found the brain of fair size for imbeciles, the pons and medulla only being specially small. The cerebral vessels were thinner than natural, and there was evidence of old arteritis in some places. The majority of Mongols fell victims to the various diseases of childhood.—Dr. G. PARKER also showed an imbecile of the same type. The child, aged three and a half, had been very weak at first, and for a long time after birth was unable to raise the head or sit up; but now had improved, and was of normal size and weight. Nystagmus was present. It was remarked that reports upon the efficacy of the thyroid treatment of cases of this type were very contradictory, some stating this treatment was absolutely useless, others that it was of considerable use if given early enough. His own case had been treated in that way, having taken grs. v. of thyroid extract twice a day. There had been much improvement, possibly due to the extract. He thought that the dwarfs of mediæval history described as having agreeable characters were of the Mongolian type, and they were of quite a different class to the spiteful dwarfs. The type seemed a very definite one. They usually died in early life from lung trouble.—Mr. MUNRO SMITH asked if these dwarfs lived to twenty-one and over. He also wished to know what dwarfs Dr. Parker had referred to as being described in history. Those described in the *Morte d'Arthur* were highly intelligent.

Dr. SHINGLETON SMITH described a case of bronchiectasis, in which the lung had been explored in search of pus. The patient, a woman 26 years of age, had been admitted to the Royal Infirmary in July, 1899. Seven weeks before she had been confined to bed a few days, suffering from inflammation of the right lung. Since then she had continued to expectorate very freely. She was fairly nourished, though she had lost sixteen pounds in weight in four months. The copious bronchorrhœa was increased by turning upon the left side. The secretion then ran into her mouth, and its expulsion was like vomiting. Over an area of three or four inches diameter at the right base posteriorly, there were heard cavernous breathing, pectoriloquy, and mucous rales, but no tympanitic resonance. The finger ends were clubbed. The sputum was greenish-yellow and fairly tenacious. It contained no elastic fibres, nor tubercle bacilli. In August the area in which there was cavernous breathing had increased. She expectorated as much as half a pint at one cough sometimes, and again shortly after as much as eight ounces at once. On November 8th, as the patient was losing ground, an attempt was made by Mr. Harsant to discover and drain a cavity in the lung. A flap was reflected exposing the ninth rib, and two inches of that bone removed. Then the pleura, which was not adherent to the lung, was incised. Air rushed in freely. The lung was brought up, sutured to the chest wall, and repeatedly explored with the aspirator. No cavity or pus could be found, and the flap was replaced. The operation had no effect one way or the other on the progress of the case. The patient steadily declined, and died on January 15th. The *post-mortem* examination showed the absence of any tubercular disease. The lower lobe of the right lung showed several very large dilated bronchial tubes, with much inter-tubular fibroid tissue. The left lung showed similar changes in a less degree. Dr. Smith remarked that he had only once known a gangrenous lung-cavity become obliterated, or almost obliterated, and recovery ensue. The dilated tubes, in the case recorded, gave the same physical signs as those given by cavities amenable to surgical relief.—Mr. Harsant, commenting on the case, said that the signs of a cavity had been very marked. It seemed as if there were a superficial cavity, yet no pus was removed nor cavity found.—Dr. Watson Williams said he had seen the case, and agreed as to the signs, but had not felt that he could diagnose the existence of a cavity with any certainty. He drew attention to the frequency with which practitioners cease to treat and to regard as worth attention, cases recovering from bronchitis, and yet not infrequently such cases caused serious bronchial trouble in later life. He alluded to the way in which gonorrhœa had formerly been regarded as quite done with when the discharge ceased. Now more serious views were taken of the affection.—Dr. J. O. Symes remembered examining the sputum in the case described, and that he had found a preponderance of streptococci and leptothrix forms. Such forms might be taken as pointing to the origin of the secretion in dilated tubes.—Dr. Michell Clarke alluded to the important practical point that if you have consolidation and signs of a cavity at the base of a lung, and no signs elsewhere in the lungs, the disease was not tubercular. Exceptionally there might be a cavity at the base, with slight signs at one apex, in tubercular disease, but it was the rarest thing to meet with tubercular disease limited to the base of the lung.—Dr. Shingleton Smith, in replying, said that he knew of several cases in which dilated tubes at the bases had given signs of cavity, and in which attempts to drain had failed. He agreed with Dr. Clarke as to the cavities at the base not being tubercular unless there were signs of the disease elsewhere.

Mr. W. Roger Williams read a paper on "The Pathogenesis of

Myoma of the Uterus." He alluded to the two distinct view held by different authorities upon the pathogenesis of tumours. According to one view they resulted from modifications in formative processes, according to the other they were modifications of inflammatory processes. The causes were therefore intrinsic according to the first view, or extrinsic according to the other. He himself believed in the intrinsic origin of tumours, and would endeavour to show his reason. Numerous observations were quoted showing that fibro-myomata were associated with other abnormalities of the reproductive organs. Their association with *uterus duplex* and *unicornis*, and with vaginal deformities was alluded to. Also that with ovarian cysts. It had been estimated that 18 % of ovarian cysts were concurrent with fibro-myomatous disease of the uterus. There were many examples of the co-existence of these tumours in the uterus, ovaries, and even in the vagina. Fibro-myomata were also associated with less obvious abnormalities. Hidden abnormalities had been found in association with these tumours whenever specially looked for. Cohnheim, Paget and others had previously noted the development of tumours at the seat of abnormal development, *e.g.*, in moles. Researches seemed to show that fibro-myomata sprang from muscular remnants of the Wolffian and Müllerian ducts. In fibroids there had been found collections of cells, and structures not unlike utricular glands, even with ciliated cells. They were believed to be Wolffian or Müllerian remnants, but they might be secluded sequestra of the uterine mucosa. Probably similar conditions might arise in post-embryonic life. During pregnancy there was a tendency to ingrowth and sequestration. Bacteria had been found in fibroids. The proneness to fibrification and calcification was due to the sequestration of germs likely to degenerate in this way.—Dr. Aust Lawrence said he thought that one-third of his ovarian cases had been associated with fibroids of the uterus. He had seen the removal of one cyst cause the diminution in size of an associated fibroid. He mentioned a case of sloughing fibroid of the vagina he had seen, in which there were also uterine fibroids present.—Dr. Walter Swayne thought that inflammatory processes had much to do with the production of tumours. He alluded to a patient he had attended for eight years suffering with endometritis. At the end of that time she developed a fibroma in the vagina. This he had removed, opening up the utero-vesical pouch. In this case, perhaps, inflammation had caused the growth of developmental relics. He believed enlarged prostate was the result of irritation, and the analgous enlargement of the uterus from fibroids might be due to irritation.—Mr. Roger Williams, in reply, reiterated his view that extrinsic causes were not primarily or chiefly concerned with the origin of tumours, any more than they were with the development of a tooth or hair.

J. Lacy Firth, *Reporter.*
J. Paul Bush, *Hon. Sec.*

---

### Plymouth Medical Society.

*October 7th, 1899.*

Mr. M. H. Bulteel, President, in the Chair.

The following office-bearers were elected for the following year :—
*President and Hon. Treasurer:* Mr. J. E. Square.
*Hon. Librarian:* Mr. C. E. Russel Rendle.
*Hon. Secretary:* Mr. W. L. Woollcombe.

The Hon. Treasurer's Report showed a balance of £33 10s., as against £11 10s. at last Annual Meeting.

A communication from the Medical Guild of Manchester *re* a proposed Medical Conference was referred to the Ethical Committee.

After the meeting twenty-five members and guests dined together.

---

*October 28th, 1899.*

Mr. J. E. Square, President, in the Chair.

Mr. S. G. Vinter, Staff-Surgeon W. M. Craig, R.N., and Mr. T. Noy Leah were elected members.

Dr. L. E. Fox showed a man, æt. 60, who had fallen and fractured the base of his skull, and subsequently developed a peculiar gait and speech.—Mr. G. F. Aldous demonstrated a phonographic reproduction of the man's voice.

Mr. W. L. Woollcombe showed a man, æt. 35, whose left upper jaw he had removed in August last by Fergusson's method, after a preliminary tracheotomy. He was now wearing a vulcanite plate with teeth, and his speech was almost natural; there was no dropping of eye or deformity of face.

Mr. C. E. Russel Rendle showed a man, æt 25, who fell from a second-story window five months ago, and cut his eyebrow, but did not lose consciousness. The right eye was closed for three weeks by swelling of the lids; when the swelling subsided there was very little sight, and even that has gradually gone. Now right eye barely p.l. Left eye, $V = \frac{6}{9}$. The right optic disc is atrophied, and there is a small retinal hemorrhage in the left eye.—Mr. Rolston pointed out that the case was similar to a number described by S. Snell, in which the lesion was proved to be between the optic commissure and the entrance of the retinal vessels to the optic nerve.

The Ethical Committee reported that after a detailed consideration of the circular presented by the Manchester Medical Guild *re* proposed Medical Conference, they had adopted the following resolution: "That this Sub-committee approves the objects of the proposed Conference, and recommends the Plymouth Medical Society to co-operate as far as possible."—The Report was received and adopted.

Dr. H. Webber showed a portion of intestine (ileum), two feet long, with mesentery all becoming gangrenous from thrombosis of mesenteric vessels. The specimen was from a man, æt 48, who died on fifth day of symptoms which were those of a gradually increasing obstruction. The abdomen was opened on fourth day, and a loop of bowel drawn out and opened.—Mr. Woollcombe thought drainage no good in these cases, and that excision of the whole thrombosed area gave the only chance of recovery if obstruction once became marked.

Mr. F. E. Raw read notes on a case of ovariotomy in which a very large cyst was removed, with considerable hemorrhage from adhesions, and in which the resulting collapse was arrested by saline injections into veins and peritoneal cavity.

Mr. Woollcombe showed a cyst of the paroöphoron removed from a woman, æt. 32, who had a large multilocular ovarian cyst, holding about six pints, in connection with the right ovary, and the specimen with the left. The specimen showed well how the cyst had distended the meso-salpinx and stretched the Fallopian tube, pushing the vertical tubes of the parovarium to the uterine side, and leaving the ovary proper attached to its lower and inner side unaltered. On the interior of the cyst-wall was a small cluster of warts characteristic of these cysts.

Mr. Hamilton showed a cast of the uterus believed to be the product of conception.

---

*November 15th, 1899.*

Mr. J. E. Square, President, in the Chair.

Mr. G. Jackson was nominated as a delegate from the Society to attend the proposed Medical Conference.

Mr. R. H. Lucy opened a discussion on "Abdominal Pain: its Varieties, and their Value as Diagnostic Aids." The discussion was carried on by several members.

---

*December 2nd, 1899.*

Mr. J. E. Square, President, in the Chair.

Mr. C. H. Whiteford showed a boy, æt. 7 years, who, twelve months ago, developed a Hunterian chancre on the left upper eyelid. He subsequently developed mucous tubercles; but under treatment the sore healed, and no other symptoms developed.

Dr. L. E. Fox read notes of (i) A Case of Congenital Cerebellar Ataxy; (ii) A Case of Pseudo-Hypertrophic Paralysis.

Mr. J. R. Rolston showed a man who, four months ago, fell out of a waggon, struck his head, and subsequently developed paralysis of the left sixth nerve.

Mr. C. H. Whiteford showed (i.) A metal horseshoe designed to pass through an opening in the mesentery and support the gut after enterostomy, instead of using a straight rod, which is apt to cause trouble from lying against the iliac crest. (ii.) An automatic wound retractor, in which the retraction was made by means of a suture in either edge of the wound, drawn on by a guy-rope which was passed beneath the table.

Dr. Fox showed a specimen of a tumour of the pons, and read notes of the case.

Walter L. Woollcombe, *Hon. Sec.*

